# The correlation between serum asprosin and type 2 diabetic patients with obesity in the community

**DOI:** 10.3389/fendo.2025.1535668

**Published:** 2025-05-05

**Authors:** Dong Liang, Xiaoyu Li, Chenyu Xiang, Minggang Xu, Yi Ren, Fen Zheng, Lijing Ma, Jing Yang, Yan Wang, Linxin Xu

**Affiliations:** ^1^ Department of Endocrinology, First Hospital of Shanxi Medical University, Shanxi Medical University, Taiyuan, Shanxi, China; ^2^ First Clinical Medical College, Shanxi Medical University, Taiyuan, Shanxi, China; ^3^ Academy of Medical Science, Shanxi Medical University, Taiyuan, Shanxi, China; ^4^ Department of Endocrinology, Changzhi Second People’s Hospital, Changzhi, China; ^5^ Shanxi Innovation Center for Integrated Management of Hypertension, Hyperlipidemia and Hyperglycemia Correlated with Cardiovascular and Cerebrovascular Diseases, Taiyuan, China; ^6^ Clinical Research Center For Endocrine and Metabolic Diseases Of Shanxi Medical University, Taiyuan, China

**Keywords:** type 2 diabetes mellitus, asprosin, obesity, community, adipokine

## Abstract

**Aims:**

Asprosin is a newly discovered adipokine, it is associated with the insulin resistance, lipid metabolism disorder, diabetes and obesity. However, there have been no reports on the relationship between asprosin and type 2 diabetic patients with obesity. This study aims to investigate the relationship between serum asprosin and type 2 diabetic patients with obesity in the community.

**Materials and methods:**

A total of 491 patients with Type 2 Diabetes Mellitus (T2DM) were recruited from Zhuoma Community Care Station and Chengbei West Street Community Care Service Center in Changzhi City of Shanxi Province from November 2019 to July 2021. Patients were divided into the Normal group (n= 145), Overweight group (n=201) and Obesity group (n= 145). The t-test, Mann–Whitney U test, and χ² test were used to compare indicators between the Normal group, Overweight group and Obesity group. Pearson or Spearman correlation analysis was adopted to evaluate the correlation between serum asprosin and other clinical data. Multivariate logistic regression analysis was applied to analyze the influencing factors on obesity. In addition, the diagnostic ability of asprosin to detect type 2 diabetic patients with obesity was tested using receiver operating characteristic (ROC) curve analysis.

**Results:**

Compared with patients with normal weight, the circulating level of asprosin was significantly higher in the obese group than in the normal weight and overweight group (P <0.05). Asprosin was positively correlated with Systolic Blood Pressure(SBP), Body Mass Index (BMI), Waistline, serum uric acid (SUA), aspartate transaminase (AST), creatinine(CRE), and negatively correlated with alanine aminotransferase(ALT). In addition, serum asprosin was significantly correlated with BMI even after adjusting for age, sex, SBP and ALT, SUA, glycosylated hemoglobin (HbA1c), low-density lipoprotein cholesterol (LDL-C), high-density lipoprotein cholesterol (HDL-C), triglycerides (TG) and glomerular filtration rate (eGFR) ((P < 0.05). Compared with the patients in the lowest tertile of serum asprosin (<250.7 pg/mL), patients with asprosin between 250.7–314.0 pg/mL [OR (95% CI) is 1.774 (0.978-3.218), P < 0.05] and asprosin >314 pg/mL [OR (95% CI) is 8.406 (4.788-14.758), P < 0.05] have a higher risk of obesity.

**Conclusions:**

Serum asprosin was correlated with obesity in community-based type 2 diabetic patients with obesity. Additionally, the risk of obesity was obviously increased with the raise of asprosin. Therefore, we speculate that serum asprosin could be used as a risk predictor of type 2 diabetic patients with obesity.

## 1 Introduction

As a global epidemic, the incidence of obesity has been steadily increasing in recent years. At present, more than 2.1 billion people in the world are overweight or obese (1). Scientific studies have confirmed that obesity is the starting point of various metabolic related diseases such as T2DM, Polycystic Ovary Syndrome (PCOS), and cardiovascular diseases (2, 3). Data shows that about 60% of type 2 diabetic patients were obese (BMI>30kg/m^2^) and combined with insulin resistance (4). Obesity could lead to T2DM through insulin resistance, which in turn could aggravate obesity and increase blood sugar (4, 5). Obesity and T2DM are cause-and-effect to each other, resulting in a vicious cycle that ultimately increases the incidence of diabetes complications and increases the social and economic burden. Therefore, the method of treating obesity and T2DM is urgent.

In recent years, domestic and foreign studies have found asprosin, a new discovered fasting-induced glucogenic adipokine, was significantly elevated in the patients with obesity and T2DM (including newly diagnosed and existing patients) (6–13). As clinical studies suggested that asprosin may be an early indicator of glucose, lipid impairment and insulin resistance. It can be used as a factor to predict the risk of T2DM and obesity (14). Furthermore, animal research indicated asprosin antibody can reduce blood sugar and appetite, suggesting that it can be used as a therapeutic target for T2DM and obesity. However, this adipokine has not been studied in people with T2DM and obesity at the same time. The purpose of this study was to explore the role of asprosin in patients with T2DM combined with obesity, and to provide new ideas for therapeutic targets for these patients.

## 2 Materials and methods

In our cross-sectional study, comprehensive clinical data of patients diagnosed with T2DM were retrospectively collected from a community health care center (Zhuoma Community Care Station and Chengbei West Street Community Care Service Center in Changzhi City of Shanxi Province) in southeastern Shanxi Province from November 2019 to July 2021. Patients who met the WHO diagnostic criteria for T2DM in 1999 (15) and had available clinical and asprosin data were included, while those with type 1 diabetes mellitus, diabetic ketoacidosis and diabetic hypertonic coma, severe liver and kidney dysfunction, serious infection, malignant tumors, communication disorders such as mental diseases or inability to cooperate with researchers, or diabetes caused by other endocrine diseases were excluded. A total of 491 subjects with T2DM were categorized into three groups based on BMI (normal weight group: BMI 19-23.9 kg/m2; overweight group: BMI 24-27.9kg/m2; obesity group: BMI≥28 kg/m2) (16), 60.1% were men(295/491). The research protocol is applicable by the ethics committee of the First Hospital of Shanxi Medical University (No. 2019 [K056]). All study participants provided informed written consent. The study kept patient data confidential and complied with the Declaration of Helsinki. There is no conflict of interests among all authors.

### 2.1 Data collection

The waist circumference, systolic blood pressure and diastolic pressure of the patients were collected, weight and height were measured to calculate BMI. Hypertension was diagnosed according to the Chinese Guidelines for the Prevention and Treatment of Hypertension (2010) criteria: systolic blood pressure ≥140 mmHg and/or diastolic blood pressure ≥90 mmHg. Fasting blood samples were collected after an overnight fast for measurement of fasting blood glucose (FPG), total cholesterol (TC), LDL-C, HDL-C, TG, CRE, HbA1c, AST, ALT, Fasting C-Peptide (FC-P), SUA and eGFR, as well as serum asprosin levels.

### 2.2 Grouping and detection of serum asprosin

Blood samples for asprosin measurement were collected and centrifuged at 3,000 rpm with a radius of 13.5 cm for 15 minutes. The resulting supernatant was then stored at -80°C. Serum asprosin levels were measured using an enzyme-linked immunosorbent assay (ELISA) with a ready-to-use detection kit provided by Herb (Shanghai Biotechnology Co., Ltd, Shanghai, China). The kit’s standards included an inter-batch difference of <11% and an intra-batch difference of <8%, with all samples being tested in duplicate wells.

### 2.3 Statistical analysis

The data were analyzed using SPSS 22.0 (International Business Machines Corporation, Amonk, NY, USA), Graphs are created by Prism 8.0 (GraphPAD software) and Q-Q plots were used to test for normality. The data are presented as mean ± standard deviation (mean ± SD) when consistent with a normal distribution, while non-normally distributed data are shown as median (range). Categorical data are presented as frequency (constituent ratio or percentage). Statistical differences in quantitative data were compared using analysis of variance or the Kruskal–Wallis H-test, and categorical data were analyzed using the Chi-square test or Fisher exact test. The relationship between serum asprosin and other participant variables was assessed using Pearson correlation coefficient analysis when the data met bivariate normal distribution; otherwise, the Spearman correlation coefficient was applied. Multivariate stepwise linear regression was conducted to analyze the correlations of asprosin with BMI. Three models were used for the multivariate logistic regression analyses: Model 1 included no adjusted variable; Model 2 adjusted for age, sex and SBP; Model 3: Model 2 plus duration of diabetes, ALT, SUA, HbA1c, LDL-C, HDL-C, TG and eGFR. Logistic regression analyses were used to determine factors independently related to in obese patients with T2DM. In addition, the multivariate logistic regression analysis used the ‘enter’ method (i.e., all factors entered the logistic regression equation at the same time), through which 95% confidence intervals (95% CIs) odds ratios (ORs) were calculated. Participants without Obesity were defined as 0 and those with Obesity as 1. Serum asprosin tertile ranges were defined as follows: tertile 1<275.25 pg/mL; tertile 2 275.25–355.08 pg/mL; tertile3>355.08pg/mL. P < 0.05 was considered statistically significant. The ROC curve was calculated to test the ability of asprosin to predict the severity of obesity in type 2 diabetic patients. Statistical significance was set at P < 0.05.

## 3 Results

### 3.1 Characteristics of participants in different groups

**Figure 1:**
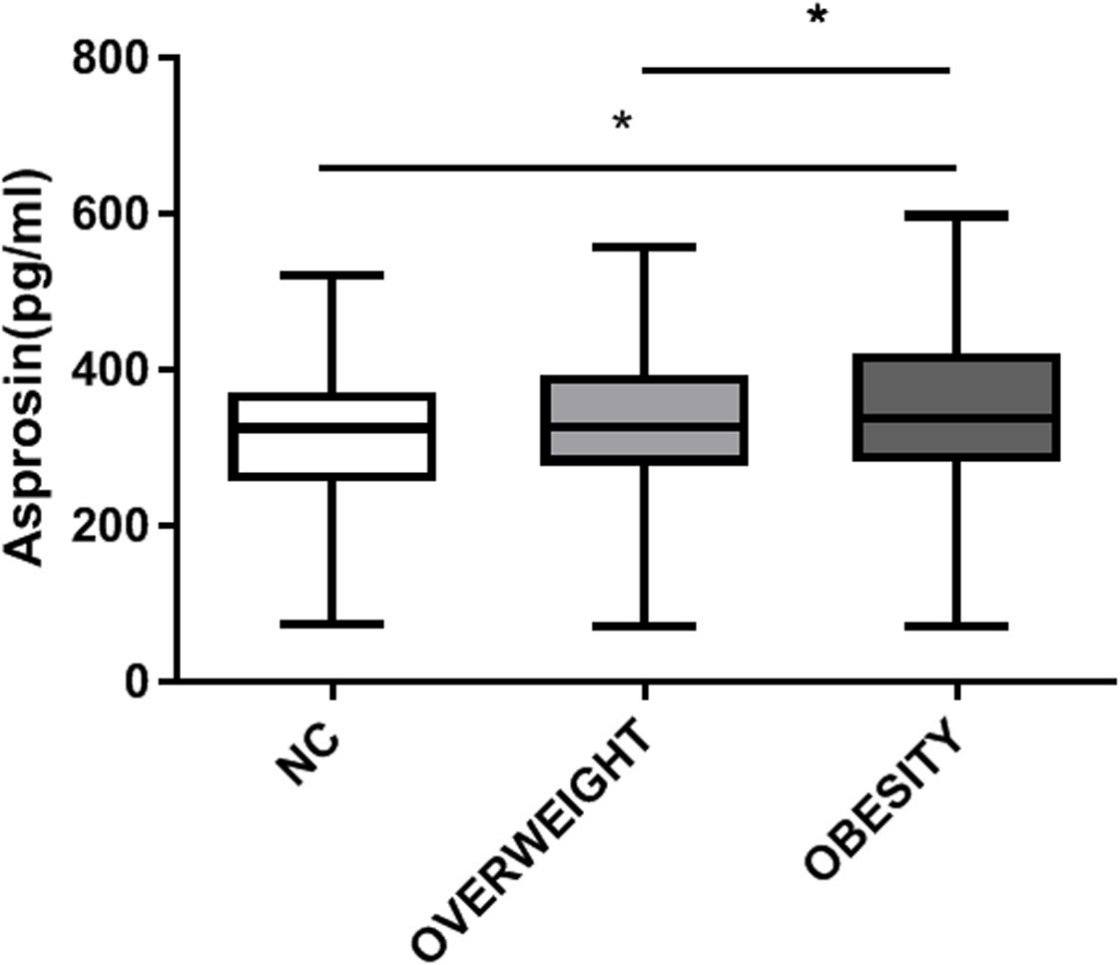
Circulating level of asprosin in each group.

A total of 491 patients with T2DM were studied, 145 with normal weight, 201 with overweight and 145 with obesity. Compared with the normal weight group, with the increase of weight, parameters including BMI, waist circumference, AST, ALT, TG, FC-P, SUA and CRE also raised in the overweight and obese groups (P <0.05) (**Table 1**), while the age and HDL-C decreased (P < 0.05). Furthermore, the circulating level of asprosin was significantly higher in the obese group than in the normal weight and overweight group (P <0.05) (**Figure 1**). In addition, according to the tertiles of serum asprosin, patients in the high-level asprosin subgroup had higher BMI, waist, SBP, TG, SUA, and FC-P than other subgroups (P<0.05). Detailed characteristics of the included patients were shown in **Table 2**.

**Table 1:** Comparison of the clinical characteristics of patients with T2DM in the community between those with Normal, Overweight and Obesity.

Characteristics	Normal (n= 145)	Overweight (n=201)	Obesity (n= 145)	*P* for trend
Age (y)	60±13	59±12	54±15	<0.001
*Duration (y)	12 .0 (7.5,18.5)	12 .0 (5.0, 17.0)	10 (4.0,15.0)	0.111
BMI (kg/cm^2^)	21.87±1.38	25.82±1.14	31.13±2.75	<0.001
Waist (cm)	85.21±8.08	95.89±6.94	107.06±9.90	<0.001
SBP (mmHg)	133±19	138±20	135±17	0.075
DBP (mmHg)	77±11	80±11	83±11	0.106
*AST (IU/L)	16 (13,21)	17 (14,23)	22 (16,31)	<0.001
*ALT (IU/L)	13 (10,19)	17 (12,25)	24 (16,38)	<0.001
*TG (mmol/L)	1.23 (0.92,1.73)	1.85 (1.33,2.70)	1.95 (1.41,2.76)	<0.001
TC (mmol/L)	4.50±1.04	4.74±1.21	4.56±1.31	0.163
HDL-C (mmol/L)	1.04±0.25	0.94±0.21	0.91±0.21	<0.001
LDL-C (mmol/L)	2.71±0.87	2.74±0.89	2.59±0.78	0.255
FPG (mmol/L)	8.04±2.81	8.56±3.24	8.71±3.07	0.143
FC-P (nmol/L)	1.55±0.96	2.19±1.22	2.50±1.28	<0.001
HbA1c (%)	9.15±2.02	9.09±2.15	9.23±2.09	0.817
UA (µmol/L)	299.03±89.31	342.92±87.41	371.15±86.74	<0.001
* CRE (µmol/L)	63 (54,75)	66 (56,83)	67 (58,87)	0.035
eGFR (mL/min/1.73 m^2^)	146.87±32.18	138.89±29.63	139.21±37.24	0.054
Asprosin (pg/ml)	249.08±58.70	269.01±66.69	348,31±97.01	<0.001

BMI, Body Mass Index; SBP, Systolic Blood Pressure; DBP, Diastolic Blood Pressure; AST, Aspartate Aminotransferase; ALT, Alanine Aminotransferase; TG, triglycerides; TC, total cholesterol; HDL-C, high-density lipoprotein cholesterol; LDL-C, low-density lipoprotein cholesterol; FPG, fasting blood glucose; HbA1c, glycosylated haemoglobin; CRE, creatinine; eGFR, estimated glomerular filtration rate; F-CP, fasting C-peptide. * means there was statistical difference between normal and obesity, overweight groups.

**Table 2:** Characteristics of patients with type 2 diabetes in the community stratified according to serum asprosin tertile.

Characteristics	Asprosin T1 (n= 156)	Asprosin T2 (n= 174)	Asprosin T3(n=161)	*P* for trend
Age (y)	58±12	58±14	58±14	0.977
*Duration (y)	10 .0 (5.0,17.0)	11 .0 (4.0, 16.3)	13.0 (7.0,17.0)	0.557
BMI (kg/cm^2^)	24.78±2.94	25.38±3.79	28.53±4.15	<0.001
Waist (cm)	92.68±10.25	93.89±11.43	101.68±11.53	<0.001
SBP (mmHg)	130±16	137±21	140±19	<0.001
DBP (mmHg)	77±11	81±11	82±12	0.001
*AST (IU/L)	17.0 (13.3,22.8)	18.0 (14.0,23.3)	19.0 (14.0,26.0)	0.356
*ALT (IU/L)	16.5 (12.0,25.8)	16.0 (11.0,27.0)	20.0 (13.0,31.5)	0.974
*TG (mmol/L)	1.48 (1.02,2.27)	1.68 (1.21,2.62)	1.80 (1.33,2.45)	0.030
TC (mmol/L)	4.46±1.03	4.75±1.22	4.63±1.30	0.089
HDL-C (mmol/L)	0.96±0.22	0.98±0.24	0.93±0.22	0.100
LDL-C (mmol/L)	2.64±0.84	2.78±0.88	2.64±0.82	0.206
FPG (mmol/L)	8.29±3.30	8.33±2.91	8.73±3.01	0.361
FC-P (nmol/L)	1.89±1.09	2.08±1.30	2.30±1.25	0.011
HbA1c (%)	8.91±2.11	9.34±2.11	9.18±2.05	0.172
UA (µmol/L)	314.40±76.78	337.72±99.65	362.06±91.28	<0.001
* CRE (µmol/L)	66.0 (56.3,79.8)	63.0 (55.0,78.0)	69.0 (58.5,89.0)	0.382
eGFR (mL/min/1.73 m^2^)	144.50±31.62	143.02±31.35	136.46±35.33	0.066

BMI, Body Mass Index; SBP, Systolic Blood Pressure; DBP, Diastolic Blood Pressure; AST, Aspartate Aminotransferase; ALT, Alanine Aminotransferase; TG, triglycerides; TC, total cholesterol; HDL-C, high-density lipoprotein cholesterol; LDL-C, low-density lipoprotein cholesterol; FPG, fasting blood glucose; HbA1c, glycosylated haemoglobin; CRE, creatinine; eGFR, estimated glomerular filtration rate; F-CP, fasting C-peptide. * means there was statistical difference between each group.

**Figure 2:**
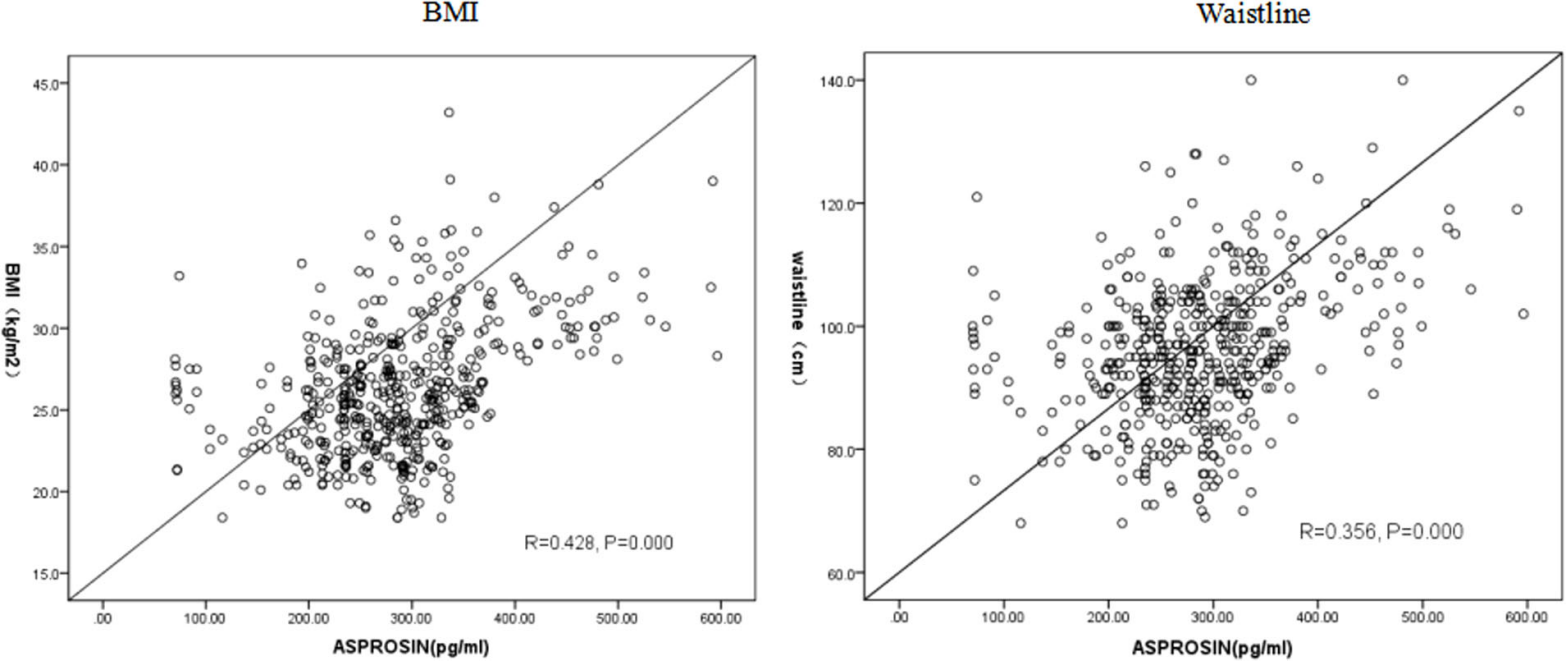
Relationship between asprosin and BMI, waistline.

Pearson correlation analysis revealed a positive association between serum asprosin level and waistline, BMI, SBP, DBP, SUA, CRE, TG, F-CP, AST, ALT (P < 0.05). Multiple linear regression analysis further demonstrated that SBP, BMI, waistline, SUA, ALT, AST and CRE were independently correlated with asprosin. Specifically, there was a positive correlation between asprosin and SBP, BMI,waistline, SUA, AST,and CRE; while a negative correlation was observed with ALT(**Table 3**). In conclusion, serum asprosin exhibited a significant positive association with BMI (**Figure 2**) and waistline (**Figure 2**) (r=0.428, P<0.05;r=0.356, P<0.05).

**Table 3:** Association between serum asprosin and other covariates in patients with type 2 diabetes.

Covariates	Pearson correlation		Multiple Linear Regression	
	*r*	*P*	*Standardized β*	*P*
*Duration (y)	0.047	0.298	–	–
Age (y)	-0.030	0.503	–	–
SBP (mmHg)	0.214	0.000	0.178	0.000
DBP (mmHg)	0.164	0.000		
BMI (kg/m2)	0.428	0.000	0.450	0.000
Waist (cm)	0.356	0.000	0.356	0.000
TC (mmol/l)	0.052	0.250	–	–
TG* (mmol/l)	0.122	0.007		
LDL- C(mmol/l)	0.008	0.868	–	–
HDL- C(mmol/l)	-0.057	0.206	–	–
SUA (µmol/l)	0.208	0.000	0.102	0.026
FPG (mmol/l)	0.034	0.456	–	–
F-CP ( nmol/L)	0.092	0.043	-0.100	0.024
HbA1c (%)	0.066	0.141	–	–
AST* (IU/L)	0.126	0.005	0.161	0.026
ALT* (IU/L)	0.101	0.025	-0.261	0.000
CRE* (umol/l)	0.099	0.028	0.213	0.000
eGFR (mL/min/1.73 m2)	-0.118	0.009		

*Spearman correlation analysis was used for skewness distribution.

BMI, Body Mass Index; SBP, Systolic Blood Pressure; DBP, Diastolic Blood Pressure; AST, Aspartate Aminotransferase; ALT, Alanine Aminotransferase; TG, triglycerides; TC, total cholesterol; HDL-C, high-density lipoprotein cholesterol; LDL-C, low-density lipoprotein cholesterol; FPG, fasting blood glucose; HbA1c, glycosylated haemoglobin; CRE, creatinine; eGFR, estimated glomerular filtration rate; F-CP, fasting C-peptide.

### 3.2 Correlation of serum asprosin levels with BMI

To further investigate the relationship between asprosin and BMI, multiple linear regression was applied (**Table 4**). The results showed that serum asprosin remained strongly correlated with BMI even after adjusting for age, sex, and SBP (P <0.05, model 2). Subsequently, additional obesity-related risk factors including ALT, SUA, HbA1c, LDL-C, HDL-C, TG and eGFR were included in the analysis. As a result, asprosin continued to show a significant association with BMI (P <0.05, model 3), indicating a close relationship between asprosin and BMI.

**Table 4:** Correlation of circulating asprosin levels with BMI in patients with type 2 diabetes.

Models	β Coefcient	95% confdence interval	*P* value
BMI			
Model 1	0.433	0.017 - 0.024	<0.001
Model 2	0.424	0.016 - 0.024	<0.001
Model 3	0.389	0.015 - 0.022	<0.001

Model 1: without adjusted variable, Model 2: adjusted for age, sex and SBP, Model 3: Model 2 plus duration of diabetes, ALT, SUA, HbA1c, LDL-C,HDL-C, TG and estimated glomerular filtration rate;sersum asprosin tertile 1, <250.7 pg/ml; sersum asprosin tertile 2, 250.7–314.0 pg/ml; sersum asprosin tertile 3, >314.0 pg/ml

### 3.3 Correlation of serum asprosin levels with the risk of obesity

The patients were subsequently categorized into three groups based on the tertiles of serum asprosin levels: low-level group (T1), middle-level group (T2), and high level group (T3). Multiple logistic regression analysis was conducted to further investigate the association between serum asprosin levels and obesity. Using T1 as the reference group, a significant increase in the incidence of obesity was observed in T2 and T3 before adjustment (Model 1) (P<0.001). After adjusting for age, sex, and SBP (Model 2), the odds ratios for T2 and T3 also increased significantly (P<0.001). Furthermore, after additional adjustments for diabetes duration, ALT, SUA, HbA1c, LDL-C, HDL-C,TG, and eGFR, it was found that compared with the T1 group, patients in the T2 and T3 groups still had a significantly higher risk of obesity (P<0.001 for both) (**Table 5**).

**Table 5:** Association of circulating asprosin levels with Obesity by logistic regression analyses in patients with type 2 diabetes.

Models	Tertile 1	Tertile 2	Tertile 3	*P* value for trend
Model 1	1	1.774 (0.978-3.218)	8.406 (4.788-14.758)	<0.001
P value		0.059	<0.001	
Model 2	1	1.915 (1.037-3.535)	10.356 (5.668-18.924)	<0.001
P value		0.038	<0.001	
Model 3	1	1.849 (0.970-3.525)	9.673 (5.127-18.253)	<0.001
P value		0.062	<0.001	

Data are presented as odds ratio (95% confidence interval) compared with tertile 1. Participants without Obesity were defined as 0 and those with Obesity as 1.Model 1: without adjusted variable, Model 2: adjusted for age, sex and SBP, Model 3: Model 2 plus duration of diabetes, ALT, SUA, HbA1c, LDL-C,HDL-C, TG and estimated glomerular filtration rate; sersum asprosin tertile 1, <250.7 pg/ml; sersum asprosin tertile 2, 250.7–314.0 pg/ml; sersum asprosin tertile 3, >314.0 pg/ml

### 3.4 ROC analysis of serum asprosin to indicate obesity for patients with T2DM

**Figure 3:**
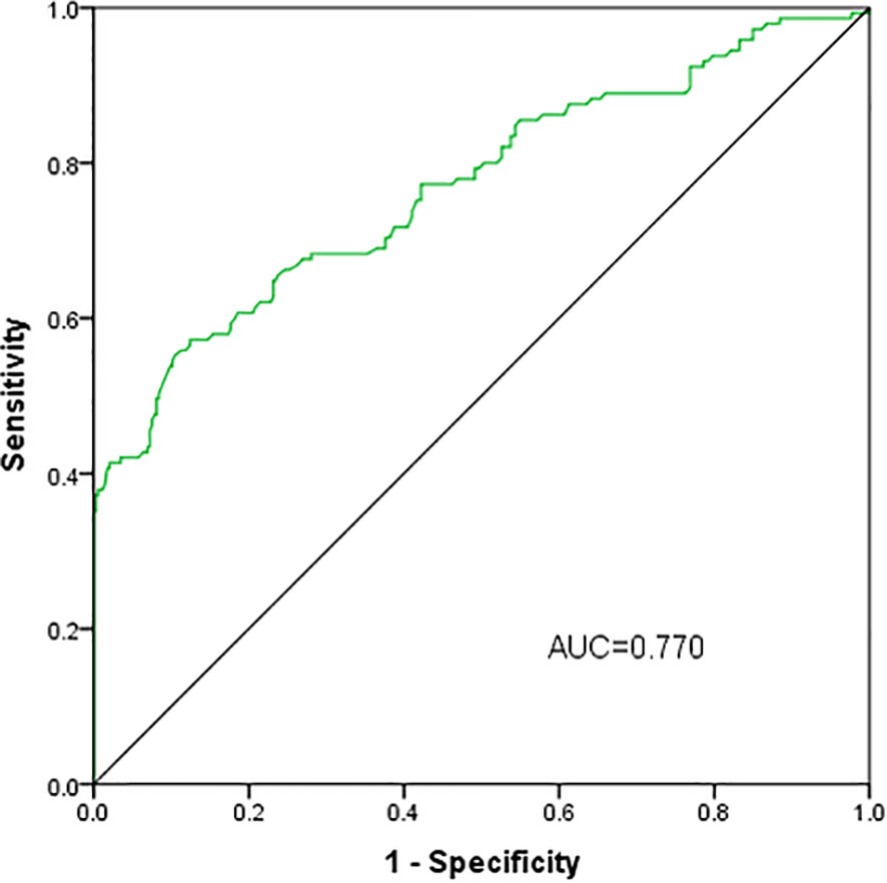
ROC analysis of serum asprosin to indicate obesity for patients with T2DM.

The predictive capacity of serum apsosin for obesity in patients with T2DM was assessed using ROC curve analysis. The area under the curve was 0.770 (95% CI: 0.720-0.820, P<0.05) with an optimal cutoff value of 332.5pg/ml achieving a sensitivity of 55.2% and specificity of 89.6%(P<0.001)(**Figure 3**).

## 4 Discussion

With the escalating prevalence of obesity, the incidence of obesity-related disorders such as diabetes increased concurrently, resulting in serious outcomes. Thus, to investigate the pathogenesis of obesity would be a great help to prevent and treat the chronic disease. Studies confirmed that adipose tissue is served as an energy storage organ and regulate various aspects of human metabolism including glucose and lipid homeostasis, insulin sensitivity and inflammatory response by secreting a variety of adipokines (14). Imbalances between pro-inflammatory and anti-inflammatory adipokines can result in metabolic dysfunctions associated with obesity, such as type 2 diabetes mellitus (T2DM) and subsequent cardiovascular diseases (17). Consequently, understanding the mechanisms underlying adipokine activity and restoring their balance may provide therapeutic avenues for addressing obesity and its related complications.

Asprosin is a recently discovered adiponectin factor secreted by white adipose tissue. As a pro-inflammatory adipocytokine, asprosin could lead to the inflammation, dysfunction and apoptosis of islet βcell through TLR4/JNK-mediated signaling in animal studies (18). It could further promote β cell apoptosis by inhibiting the autophagy of β-cell via AMPK-mTOR pathway (19). Besides, clinical trials suggested that pathological elevated asprosin levels were observed in some metabolic diseases, such as obesity, T2DM, diabetes complications, and PCOS (14).

Pre-clinical and clinical research has indicated that asprosin may serve as an independent risk factor for patients with obesity and T2DM (10, 20–22). However, prior studies have primarily concentrated on isolated cases of T2DM or simple obesity, neglecting the examination of serum asprosin levels in patients who present with both conditions. In clinical practice, T2DM and obesity frequently coexist rather than manifest independently; they often exacerbate one another. Consequently, our objective is to investigate the relationship between asprosin levels and individuals suffering from both T2DM and obesity. This study aims to provide convenient and accurate predictors for this specific population while establishing a foundation for targeted therapeutic interventions.

This study confirmed that in type 2 diabetic patients, the circulating levels of asprosin were significantly higher in the obese group compared to both the normal weight and overweight groups (P < 0.05) (**Figure 1**). Furthermore, a statistically significant difference was observed in serum asprosin levels between the overweight and obese groups, with asprosin concentrations increasing alongside body weight. Additionally, serum asprosin levels exhibited positive correlations with BMI and waist circumference (r=0.428, P<0.05; r=0.356, P<0.05), which aligns with previous research on adult obesity (6–9). In contrast, studies focusing on children with obesity have produced differing conclusions. Corica (23) and Long (24) reported lower blood asprosin concentrations in obese children when compared to normal controls, noting gender differences among participants. Wang et al. (7) found no significant variation in asprosin concentration across mild, moderate, and severe obesity categories. These discrepancies may be attributed to variations in age among the child subjects studied. Thus, it is hypothesized that asprosin may serve not only as a predictor of obesity but also for assessing its severity.

Furthermore, asprosin has been confirmed to be associated with lipid metabolism (25). This study demonstrated that, compared to the normal weight group, both TG levels increased in the overweight and obesity groups as body weight rose (P < 0.05) (**Table 1**), while HDL-C levels decreased (P < 0.05) (**Table 1**). Correspondingly, an increase in asprosin was also associated with a rise in TG levels (P < 0.05), which aligns with findings from most studies (17, 20, 25–28). The results indicated that asprosin is positively correlated with TG and negatively correlated with HDL-C; however, it showed no significant relationship with LDL-C. Nevertheless, some researchers have presented differing viewpoints. Li (13) posited that serum asprosin is positively correlated with LDL-C in females, while Ugur (6) suggested that both serum and salivary asprosin are positively correlated with LDL-C. These contrasting conclusions may be attributed to gender differences and the relatively limited number of subjects involved. Similarly, another study conducted on children reported no correlation between asprosin and TC, LDL-C, or HDL-C (24), potentially due to the age factor of the participants.

Other metabolic markers were also observed in this study. According to the tertiles of serum asprosin levels, patients in the high asprosin subgroup exhibited higher SUA levels compared to those in the overweight and obese groups (p<0.05). This finding has not been previously reported, suggesting that under obese conditions, asprosin may contribute to an increased incidence of hyperuricemia in patients with T2DM through an unknown mechanism. Furthermore, our results indicated a positive correlation between asprosin and SBP, while no significant relationship was found with DBP, which aligns with conclusions drawn from other research conducted by Xu et al. (29). Regarding hepatic enzymes, our study suggested that there was no significant correlation between asprosin and ALT or AST as body weight increased. In contrast, studies by Long (24) and Liu (30) confirmed a negative relationship between asprosin and AST. The discrepancy may be attributed to the fact that subjects recruited for their research were exclusively children.

In terms of blood glucose, numerous studies have confirmed that fasting blood levels of asprosin are significantly elevated in patients with T2DM, newly diagnosed T2DM, and those with abnormal glucose tolerance when compared to individuals with normal glucose tolerance (10–13, 21). These findings suggest that asprosin exhibits a notable response to fluctuations in glucose levels among type 2 diabetic patients and may play a crucial role in both the early stages and progression of T2DM through the mechanism of insulin resistance. A study conducted on an Iraqi population (31) reported elevated blood levels of asprosin in individuals with long-standing T2DM but not in those who were newly diagnosed; this discrepancy may be attributed to racial differences. Nevertheless, our research indicated no significant correlation between asprosin levels and FPG or HbA1c, which contrasts with previous studies (10, 13, 20, 21). This difference may arise from the fact that prior investigations primarily focused on individuals with uncomplicated T2DM without obesity. Furthermore, studies examining childhood obesity revealed that unlike adults, asprosin was not correlated with insulin resistance (26).

Asprosin has emerged as a focal point in the study of diabetes and obesity. Recent research indicates that several antidiabetic medications, including metformin, acarbose, liraglutide (a GLP-1 receptor agonist), and dapagliflozin, have been shown to lower blood glucose levels while concurrently reducing asprosin levels in patients with diabetes (32–35). Furthermore, the administration of asprosin antibodies has demonstrated efficacy in decreasing serum asprosin levels and lipid profiles, along with reductions in food intake and body weight in diet-induced obese mice. This intervention exhibits relatively reliable long-term efficacy and safety (36). Consequently, asprosin may represent a promising therapeutic target; thus, investigating the relationship between asprosin and obesity concerning T2DM is essential. However, it is noteworthy that all previous investigations were conducted exclusively on individuals with either uncomplicated T2DM or simple obesity; none included subjects affected by both T2DM and obesity simultaneously. Our research concluded that serum asprosin levels are positively correlated with BMI among individuals suffering from both T2DM and obesity. Therefore, serum asprosin could serve as a potential predictor for assessing the risk and severity of obesity within this population.

However, this research has certain limitations. It is a cross-sectional study with a small sample size, and the potential causal relationship between serum asprosin and obesity in patients with type 2 diabetes requires further validation through future prospective studies, as well as animal and cellular investigations.

## 5 Conclusions

Serum asprosin levels were found to be correlated with obesity in community-based patients with T2DM in Changzhi, Shanxi Province, China. Furthermore, the risk of obesity significantly increased with rising asprosin levels. Therefore, we propose that serum asprosin may serve as a potential risk predictor for obesity in individuals with T2DM.

## Data Availability

The raw data supporting the conclusions of this article will be made available by the authors, without undue reservation.
